# Transcranial Electrical Stimulation over Dorsolateral Prefrontal Cortex Modulates Processing of Social Cognitive and Affective Information

**DOI:** 10.1371/journal.pone.0126448

**Published:** 2015-05-07

**Authors:** Massimiliano Conson, Domenico Errico, Elisabetta Mazzarella, Marianna Giordano, Dario Grossi, Luigi Trojano

**Affiliations:** 1 Neuropsychology Laboratory, Department of Psychology, Second University of Naples, Caserta, Italy; 2 Department of Neuromotor Physiology, Scientific Institute Foundation Santa Lucia, Rome, Italy; Università di Trento, ITALY

## Abstract

Recent neurofunctional studies suggested that lateral prefrontal cortex is a domain-general cognitive control area modulating computation of social information. Neuropsychological evidence reported dissociations between cognitive and affective components of social cognition. Here, we tested whether performance on social cognitive and affective tasks can be modulated by transcranial direct current stimulation (tDCS) over dorsolateral prefrontal cortex (DLPFC). To this aim, we compared the effects of tDCS on explicit recognition of emotional facial expressions (affective task), and on one cognitive task assessing the ability to adopt another person’s visual perspective. In a randomized, cross-over design, male and female healthy participants performed the two experimental tasks after bi-hemispheric tDCS (sham, left anodal/right cathodal, and right anodal/left cathodal) applied over DLPFC. Results showed that only in male participants explicit recognition of fearful facial expressions was significantly faster after anodal right/cathodal left stimulation with respect to anodal left/cathodal right and sham stimulations. In the visual perspective taking task, instead, anodal right/cathodal left stimulation negatively affected both male and female participants’ tendency to adopt another’s point of view. These findings demonstrated that concurrent facilitation of right and inhibition of left lateral prefrontal cortex can speed-up males’ responses to threatening faces whereas it interferes with the ability to adopt another’s viewpoint independently from gender. Thus, stimulation of cognitive control areas can lead to different effects on social cognitive skills depending on the affective vs. cognitive nature of the task, and on the gender-related differences in neural organization of emotion processing.

## Introduction

Social cognition refers to the different psychological processes enabling individuals of the same species to interact with one another; such interaction essentially relies on perception of “social signals”, such as facial expressions, eye gaze and body posture [1–3]. Primates, and especially humans, are able to mentally represent what might be going on in others’ minds through high-level processing of such social signals.

In recent years, many imaging studies investigated the neural correlates of understanding others’ mind, a process known as mentalizing or theory of mind [4]. Different experimental tasks (requiring judgments on facial expressions, stories or moving shapes) consistently demonstrated the activation of a set of regions that include anterior medial prefrontal cortex, posterior cingulate cortex, the superior temporal sulcus and the adjacent temporo-parietal junction [5–6]. Activity in these domain-specific neural structures devoted to processing relevant cognitive and affective cues is modulated by domain-general control areas, such as the lateral prefrontal cortex and the anterior cingulate cortex [7–9].

Recent neurofunctional studies showed that lateral prefrontal cortex is involved in cognitive control of emotional information conveyed by facial expressions [9–11] or complex scenes [12–13]. In a functional magnetic resonance imaging study, Zaki et al. [9] required participants to draw inferences about the emotional states of another person based on congruent or incongruent social cues (facial expressions and contextual signals; e.g., person’s smiling while describing an upsetting event). Results showed that conflicting social cues implied activation of the lateral prefrontal cortex and the anterior cingulate [9]. The right lateral prefrontal cortex seems to be particularly involved in processing emotional facial expressions [14–15], but the debate is open on whether a right hemispheric predominance exists for processing all emotions, irrespective of their valence [14,16], or whether the two hemispheres preferentially process different emotions, being the left hemisphere dominant for positive emotions and the right dominant for negative ones (the valence theory) [17].

A different line of research underlined the role of the lateral prefrontal cortex in cognitive control over social, non-affective situations in which one had to take another person’s point of view (visual perspective taking) [18–19]. Neuroimaging data showed stronger lateral prefrontal cortex activation for other person’s perspective compared with self-perspective judgments [20]; in particular, it has been suggested that when judging another’s perspective the dorsolateral prefrontal cortex (DLPFC) cortex inhibits one’s own perspective selectively [21]. However, in a study in which participants were required to select between competing visual perspectives [22], Ramsey et al. [18] showed that the right DLPFC is involved in selecting the appropriate viewpoint by inhibiting the irrelevant perspective in a viewpoint-independent manner. These findings are consistent with previous neurophysiological and neuropsychological evidence showing that right lateral prefrontal cortex is involved in resolving conflicts between perspectives [19,23].

From the data reviewed above it could be inferred that lateral prefrontal cortex exerts a cognitive control over both cognitive and affective facets of social cognition, but some recent neuropsychological evidence reported dissociations between these two components of social cognition [24–25]. On the same line, inhibition of right DLPFC by 1 Hz repetitive transcranial magnetic stimulation (rTMS) speeded-up response times on a task assessing cognitive mentalizing abilities, but did not significantly modify performance on an affective task [26]. This result would support the cognitive/affective dissociation in the domain of social cognition, but left open the issue of the role of lateral prefrontal cortex in mentalizing tasks.

Several lines of evidence demonstrated that these social cognitive abilities can be shaped by gender. For instance, females generally perform better (in terms of both accuracy and processing speed) than males in recognizing emotional facial expressions [27–28]. Moreover, gender seems to affect the way in which an individuals understand others’ point of view, with females employing cognitive strategies different from those adopted by males [29–30]. Although gender is recognized as a major source of individual differences in processing specific social signals, such as facial emotional expressions [31], it is still not clear whether gender also modulates domain-general control processes of social cognition. Moreover, it is worth remembering that there is some evidence showing that male and female participants react differently to electrical brain stimulation (for instance see [32–34]). Since the effects of brain stimulation are dependent on the baseline cortical activity and activation of specific neural circuits, it has been suggested that differences in gender-related patterns of baseline brain activation could partly account for different responses to tDCS in males and females [32].

On the basis of the aforementioned considerations, we aimed to clarify whether the lateral prefrontal cortex exerts domain-general or domain-specific control over processing of cognitive and affective aspects of social cognition, and whether gender can shape such control processes. To these purposes, we compared the effects of transcranial direct current stimulation (tDCS) over DLPFC in male and female healthy volunteers who were required to process social cues in two tasks: one affective task, tapping explicit recognition of emotional facial expressions, and one cognitive task, assessing the ability to adopt another person’s visual perspective. In the affective task participants had to label the six basic emotions, i.e., happiness, fear, anger, disgust, surprise and sadness, conveyed by faces from the “Pictures of Facial Affect” set [35]. In the cognitive task, participants had to explicitly code the left-right location of a target object in a scene from their own (self) or the actor’s (other) perspective [36]; the actor acted, gazed or acted/gazed toward a target object in the scene. In this task, healthy participants provide a higher number of responses anchored on the actor’s position (altercentric responses) when observing the actor grasping the target; this effect is likely related to the activation of simulation processes [36–37] that instead cannot be activated in individuals affected by autism spectrum disorders [38].

In the present study, we used a bi-frontal tDCS that can reliably modulate high-level executive control [39–40]. In particular, Fecteau et al. [39–40] showed that the bi-frontal stimulation over DLPFC significantly modified participants’ decision making behavior whereas unilateral tDCS stimulation yielded no effect. The authors suggested that tDCS exerts its strongest influence by altering the relative balance of the dorsolateral prefrontal cortices: a cross-hemisphere interplay would play a crucial role during decision making and would be interfered with by the bilateral tDCS [39–40]. The idea of cross-hemispheric interplay and of reciprocal inter-hemispheric inhibition has been proposed also to account for the effects produced by TMS stimulation over the DLPFC on theory of mind tasks [26]. Analogously, recent tDCS studies assessing the role of DLPFC on processing emotional [41] and identity [42] information conveyed by faces adopted a bi-frontal stimulation paradigm, assuming that increasing activation in one hemisphere and decreasing activation in the other can led strong behavioral effects.

Data reviewed above suggest that the right lateral prefrontal cortex is particularly involved in processing affective information and in dealing with conflicting visual perspectives. On this basis, we could hypothesize that anodal (activating) stimulation of the right hemisphere coupled with cathodal (inhibiting) stimulation of the left hemisphere has strongest modulatory effects on social cognitive and affective abilities than the opposite stimulation pattern. On the basis of evidence showing gender related differences on social cognition, we could expect a differential effect of tDCS in males and females [27–28,31], but available data do not allow to make precise predictions about its direction.

## Material and Methods

### Participants

Sixteen healthy right-handed participants (age-range 22–30; 8 male) were enrolled for the study. The participants were all right-handed, free of drugs and without any known neurological or psychiatric condition. All participants were naïve to the nature of the experiment and gave their written informed consent before taking part in the study.

The experimental protocol has been approved by the local ethical committee (“Comitato Etico del Dipartimento di Psicologia della Seconda Università di Napoli”) and conducted in accordance with the ethical standards of the Helsinki Declaration.

### Procedure

Using a randomized, cross-over design, each participant was exposed to 15 minutes of sham, left anodal/right cathodal, and right anodal/left cathodal tDCS applied at 1.0 mA [43]. The order of the three stimulation conditions was counterbalanced and randomized across participants, with a one-week rest between conditions.

The stimulation was induced with two saline-soaked surface sponge electrodes 35 cm^2^ in size, and delivered by a battery-driven, constant current stimulator (multifunctional system for low-intensity transcranial electrical stimulation, BrainSTIM). For left anodal/right cathodal tDCS, the anode electrode was placed over the left DLPFC (F3 according to the international EEG 10/20 system) and the cathode electrode was placed over the right DLPFC (F4), whereas for right anodal/left cathodal stimulation, the polarity was reversed (the anode electrode was placed over the F4 and the cathode electrode was placed over the F3). For sham stimulation, the electrodes were placed at the same positions as for active stimulation, but the stimulator was only turned on for 20 s; participants thus felt the initial itching sensation associated with tDCS but received no active current for the rest of the stimulation period.

An off-line stimulation paradigm was used since previous work suggests that effects are more robust than on-line stimulation, at least for anodal stimulation [44]. Thus, participants performed the two experimental tasks (see below), each lasting about 4–5 minutes, after the tDCS stimulation; order of the two tasks was counterbalanced across subjects and conditions.

Stimulus presentation and data collection were controlled by a PC running Cedrus SuperLab v.4. Data have been analyzed using Statistical Package for Social Sciences (SPSS 19.0). All raw data are available in S1 File.

### Experimental tasks

#### Recognition of emotional facial expressions

Stimuli were photographs (8.6° x 10.4° of visual angle at a viewing distance of 60 cm) of 10 white Caucasian individuals (5 females) displaying a happy, fearful, angry, disgusted, surprised or sad expression, selected from the classical “Pictures of Facial Affect” set [35]; hair and non-facial areas were digitally occluded so that only the central face area was visible. Stimuli were centrally presented on a computer screen until subjects gave their response. In each trial, a fixation point (800 ms) was followed by a facial stimulus that remained on the screen until subjects gave their response. The 60 experimental stimuli (10 items x 6 emotions) were presented in a random order and preceded by six practice trials consisting in pictures of one additional model posing the six emotional expressions.

For each stimulus, subjects were required to choose the expressed emotion among six labels (i.e., happiness, sadness, anger, fear, disgust and surprise). Participants used their right hand to provide their responses on a conventional keyboard by pressing one of six keys (“R, T, Y, U, I, O”) marked with the emotion labels. The starting point of the response hand was with the index finger placed on the “H” key that in the conventional keyboard is centrally located with respect to the 6 response keys; after each response, the index finger returned on the “H” key. The order of the six keys was counterbalanced across subjects in order to control for the possible effects of key location on participants’ responses. Participants were encouraged to respond as fast and correctly as possible; both Reaction Times (RTs, in milliseconds) and accuracy were recorded.

#### Visual Perspective Taking

Participants were presented with scenes (each scene enclosed in a rectangular frame of 8.2° x 6.1° of visual angle at the viewing distance of 60 cm) representing a human model (an actor) at a table on which one target object (a bottle or a glass) was positioned. Four “actor scenes” were devised. In the first scene, the actor had a straight gaze and did not grasp the target (no-gaze/no-action). In the second scene, the actor had a straight gaze but grasped the target (no-gaze/yes-action). In the third scene, the actor gazed towards the target but did not grasp it (yes-gaze/no-action), whereas in the fourth scene, the actor both gazed towards and grasped the target (yes-gaze/yes-action); in both yes-gaze conditions, the actor looked precisely at the point in which his hand (would) come into contact with the target object. In a control scene, which served for catch trials, no actor was present.

Before starting the task, subjects were presented with instructions specifying the reference frame (one’s own or the actor’s point of view) that they had to adopt for target coding. Thus, task instructions for the self-perspective were as follows: “Where is the bottle/glass? On the left or on the right with respect to your own point of view?”. For the other-perspective, instead, instructions were: “Where is the bottle/glass? On the left or on the right with respect to the actor’s point of view?”.

In each trial, a fixation point (800 ms) was followed by a visual scene that remained on the screen until subjects gave their response. The five scenes were presented 12 times in a random order, for a total of 60 trials. Participants responded by pressing one of two buttons on the computer keyboard (“B” for left and “H” for right on a conventional keyboard) with their right dominant hand. The catch trial remained on the screen for 1500 ms, and the subjects had to withdraw from responding and to wait for presentation of the next trial. Before the task, ten practice trials were given and were discarded from statistical analysis.

The subjects’ responses were transformed according to a binary code: (left or right) responses consistent with self-perspective were scored as 0, whereas (left or right) responses consistent with other-perspective were scored as 1. Statistical analyses took into account the mean proportion of participants’ altercentric responses and RTs on altercentric responses. The proportion of altercentric responses underwent a non-linear arcsine transformation to make them suitable for parametric statistical tests [36,45].

## Results

### Recognition of emotional facial expressions

Error rates underwent a three-way mixed-design Analysis of Variance (ANOVA), with emotion (disgust, happiness, fear, anger, surprise and sadness) and stimulation condition (anodal F3/cathodal F4, anodal F4/cathodal F3 and Sham) as within subject factors, and with gender (male and female) as a between subject factor. The results showed a significant main effect of gender, F(1,14) = 8.421, p = .012, η^2^_p_ = .376, with lower error rates in females (mean = .110, SEM = .093) than in males (mean = .492, SEM = .092). No other main effect or interaction was statistically significant (all p > .05).

The same three-way mixed-design ANOVA as above was performed on RTs for correct responses. The results showed significant main effects of the emotion, F(5,70) = 31.878, p = .0001, η^2^_p_ = .695, and of stimulation condition, F(2,28) = 10.311, p = .0001, η^2^_p_ = .424, whereas the main effect of gender was not significant (p > .05). Moreover, we found a significant interaction among emotion, stimulation and gender, F(10,140) = 3.541, p = .0001, η^2^_p_ = .202. No other interaction was statistically significant (p > .05).

**Fig 1:**
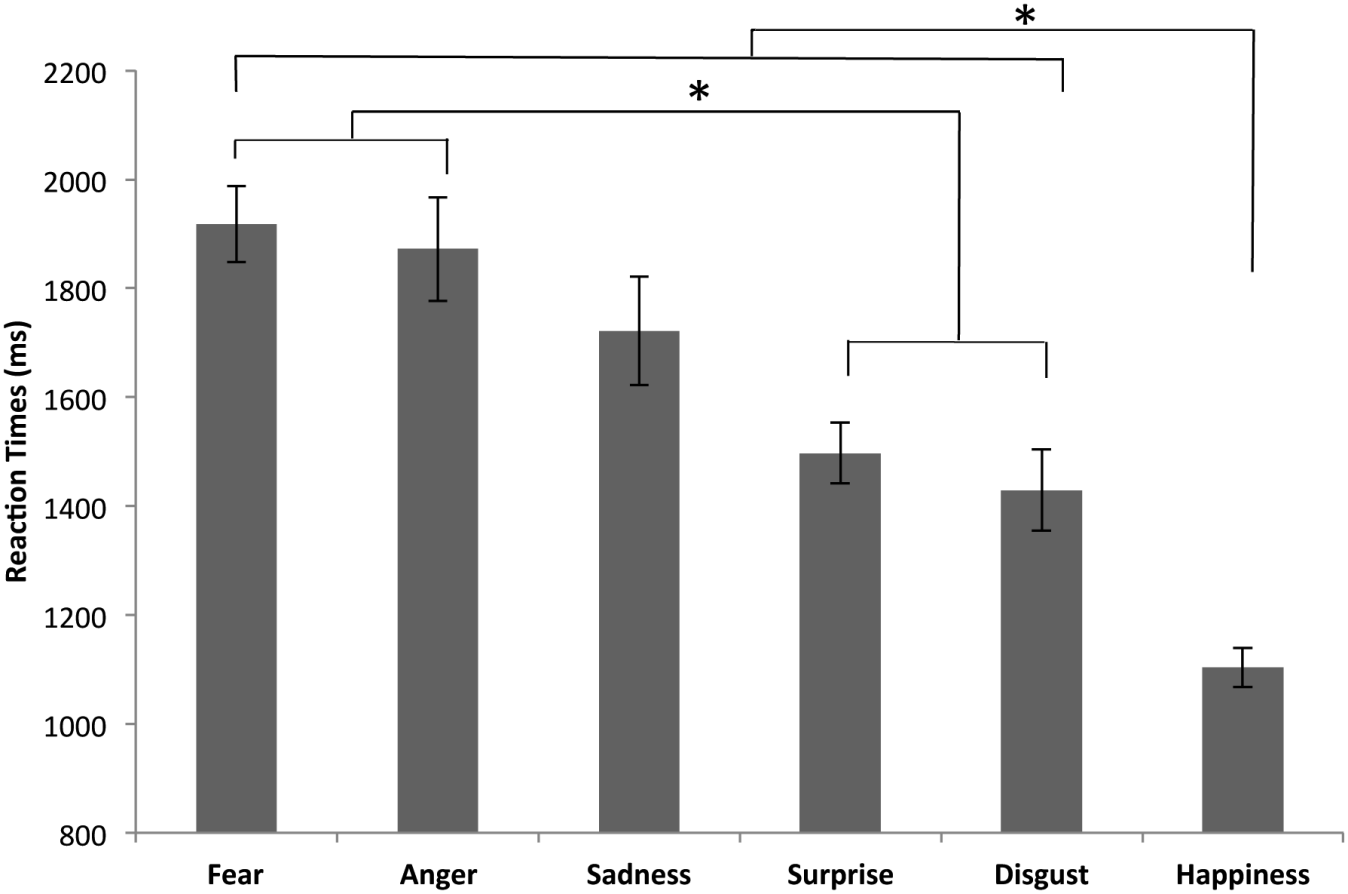
Recognition of emotional facial expressions. RTs are plotted against the six emotions (disgust, happiness, fear, anger, surprise and sadness); bars represent SEM. Significant comparisons are highlighted by an asterisk (p < .05).

Bonferroni-corrected pairwise comparisons on the main effect of emotion (Fig 1) showed that recognition of happiness was significantly faster than recognition of all the other emotions (all p < .001); recognition of disgust and surprise was faster than recognition of anger and fear (all p < .006). Bonferroni-corrected pairwise comparisons on the main effect of stimulation condition showed that RTs were significantly faster on anodal F4/cathodal F3 than on anodal F3/cathodal F4 (p = .003) and sham (p = .017), whereas there were no differences between anodal F3/cathodal F4 and sham (p = 1).

**Fig 2:**
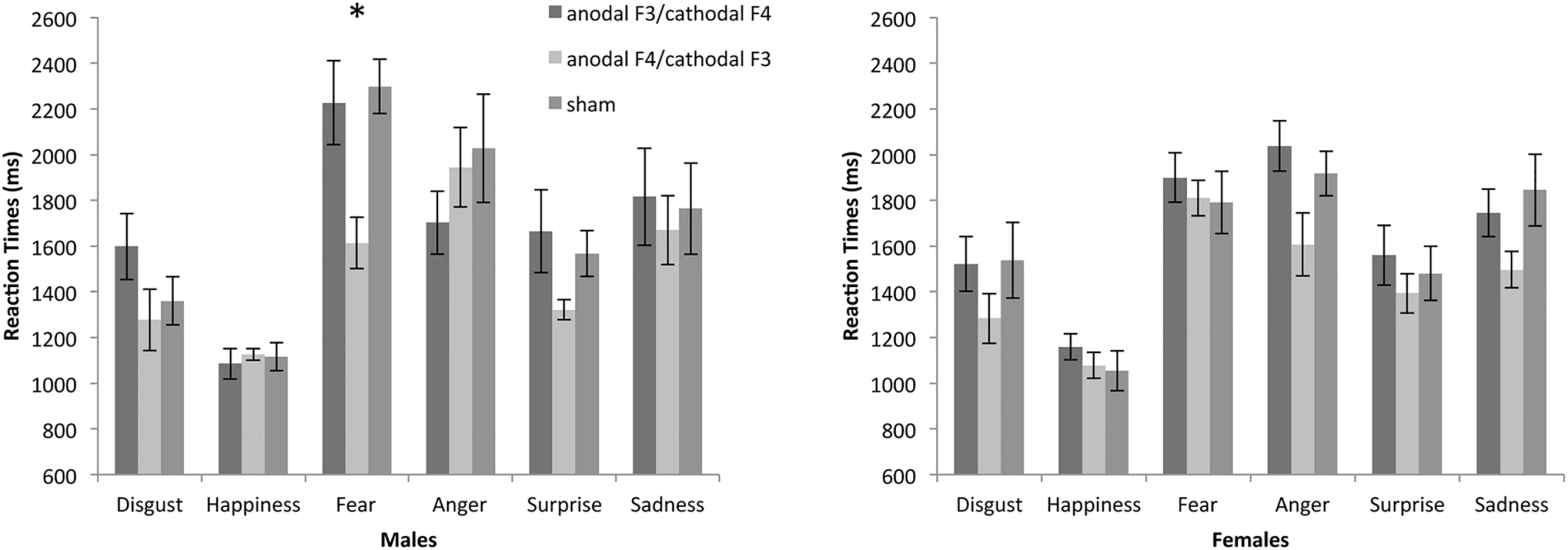
Recognition of emotional facial expressions. RTs plotted against the three stimulation conditions (anodal F3/cathodal F4, anodal F4/cathodal F3 and sham) and the six emotions (disgust, happiness, fear, anger, surprise and sadness), separately in males and females. Bars represent SEM. * Significant at p < .05.

To explore the interaction among emotion, stimulation and gender, RTs were submitted to two-way repeated measures ANOVAs, for males and females separately. Results of the ANOVA on RTs of male participants showed a significant main effect of the emotion, F(5,35) = 15.091, p = .0001, η^2^_p_ = .683, in line with results from the general ANOVA. Moreover, we also found a significant main effect of stimulation condition, F(2,14) = 5.099, p = .030, η^2^_p_ = .421, showing that RTs on anodal F4/cathodal F3 were significantly faster than sham (p = .048) but did not differ from anodal F3/cathodal F4 (p > .05). More relevant, we found a significant interaction between emotion and stimulation, F(10,70) = 4.077, p = .0001, η^2^_p_ = .368. Bonferroni-corrected pairwise comparisons on the emotion x stimulation interaction showed that when recognizing fearful expressions RTs were significantly faster on anodal F4/cathodal F3 than on anodal F3/cathodal F4 (p = .004) and sham (p = .025), whereas there were no differences between anodal F3/cathodal F4 and sham (p = 1). Post-hoc analysis of all the other combinations between stimulation condition and facial expressions did not reveal significant differences (all p > .05; Fig 2).

Results of the ANOVA on RTs of female participants showed a significant main effect of the emotion, F(5,35) = 20.226, p = .0001, η^2^_p_ = .743, in line with the results of the general ANOVA. Moreover, we also found a significant main effect of stimulation condition, F(2,14) = 6.338, p = .019, η^2^_p_ = .475, showing that RTs on anodal F4/cathodal F3 were significantly faster than anodal F3/cathodal F4 but did not differ from sham (p > .05). The interaction between emotion and stimulation was not significant (p > .05; Fig 2) differently form what found in male participants.

### Visual Perspective Taking

Arcsine-transformed altercentric responses underwent a five-way mixed-design ANOVA, with gaze (no-gaze and yes-gaze), grasping (no-action and yes-action), perspective (self and other) and stimulation condition (anodal F3/cathodal F4, anodal F4/cathodal F3 and Sham) as within subject factors, and with gender (male and female) as a between subject factor. Results showed a significant main effect of perspective, F(1,14) = 1792.661, p = .0001, η^2^_p_ = .992, with a higher number of altercentric responses in the other-perspective than in the self-perspective. We also found significant interactions between grasping and stimulation, F(2,28) = 6.167, p = .007, η^2^_p_ = .306, and among grasping, perspective and stimulation, F(2,28) = 5.475, p = .010, η^2^_p_ = .281. No other main effect or interaction was statistically significant (p > .05).

**Fig 3:**
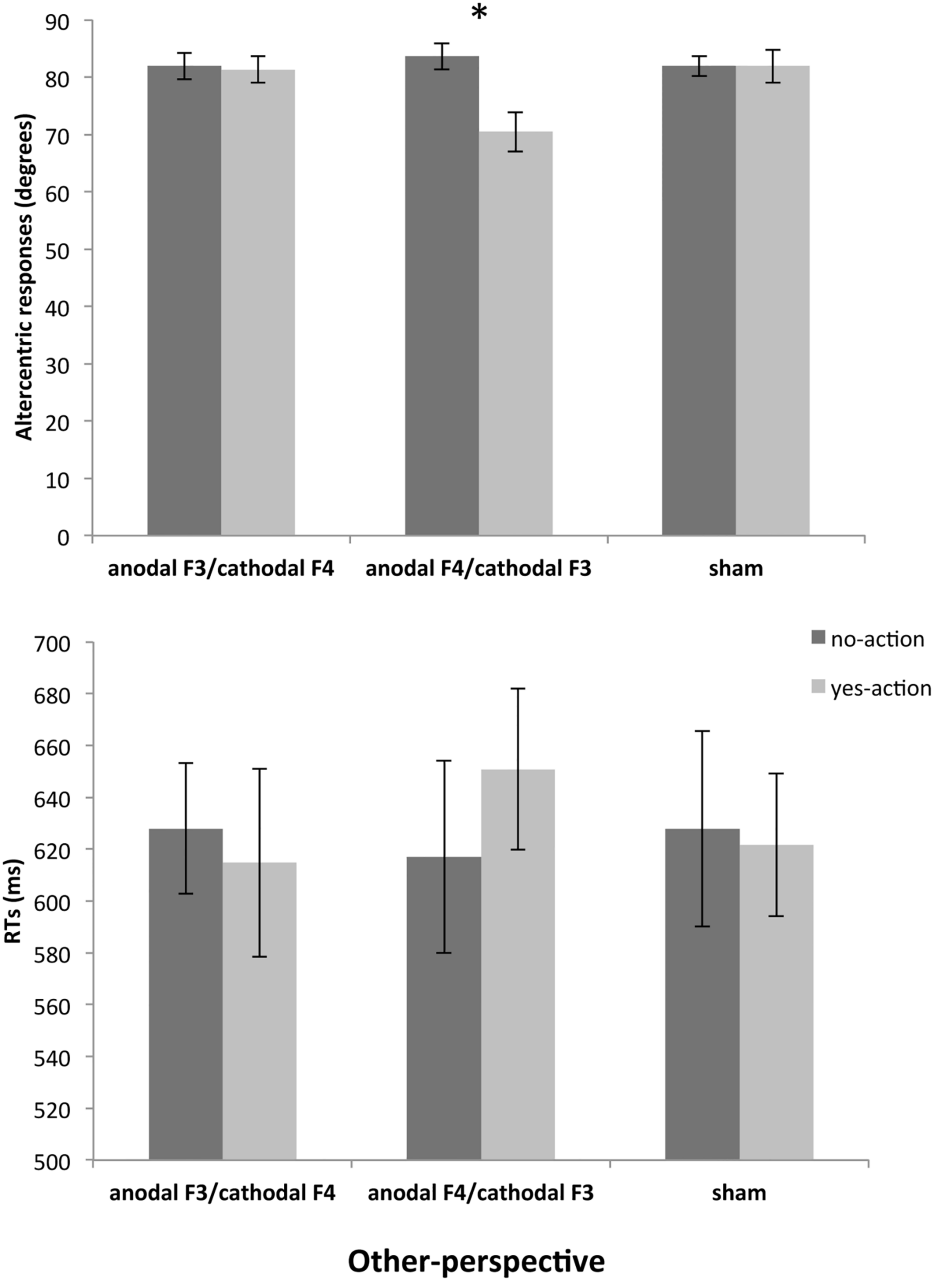
Visual Perspective Taking. Mean proportion of altercentric responses (upper panel; values on y-axis are degrees: 90 degrees correspond to 100% of altercentric responses) and RTs on altercentric responses (lower panel) plotted against the three stimulation conditions (anodal F3/cathodal F4, anodal F4/cathodal F3 and sham), separately for no-action and yes-action conditions. Bars represent SEM. *The number of altercentric responses in the anodal F4/cathodal F3 stimulation for yes-action stimuli was significantly lower with respect to no-action stimuli in the same condition (p < .001), and with respect to yes-action stimuli in the other stimulation conditions (both p > .002).

To explore the interaction among grasping, perspective and stimulation the arcsine transformed altercentric responses were submitted to two-way repeated measures ANOVAs, for self- and other-perspectives separately. ANOVA on self-perspective trials did not reveal any significant main effect or interaction (all p > .05). On the other-perspective trials ANOVA showed a significant main effects of grasping, F(1,14) = 6.475, p = .023, η^2^_p_ = .316, with more altercentric responses in the no-action than in the yes-action condition, and of stimulation, F(2,28) = 5.444, p = .018, η^2^_p_ = .280, with less altercentric responses in anodal F4/cathodal F3 than in anodal F3/cathodal F4 and sham. Moreover, we found a significant interaction between grasping and stimulation, F(2,28) = 10.709, p = .0001, η^2^_p_ = .433. Bonferroni-corrected pairwise comparisons showed a significantly lower number of altercentric responses in the anodal F4/cathodal F3 stimulation for yes-action stimuli with respect to no-action stimuli in the same condition (p < .001), and with respect to yes-action stimuli in the other stimulation conditions (both p > .002; Fig 3).

RTs for altercentric responses in other-perspective trials underwent a four-way mixed-design ANOVA, with gaze (no-gaze and yes-gaze), grasping (no-action and yes-action), and stimulation condition (anodal F3/cathodal F4, anodal F4/cathodal F3 and Sham) as within subject factors, and with gender (male and female) as a between subject factor. Results did not reveal any significant main effect or interaction (all p > .05), but it is worth noting that the pattern of results fitted that observed in the analysis on proportion of altercentric responses (see Fig 3).

## Discussion

In the present study we assessed whether cognitive control over cognitive and affective components of social cognition can be modulated by bi-hemispheric tDCS stimulation over the DLPFC. Results showed that in males, but not in females, explicit recognition of fearful facial expressions was significantly faster after anodal F4/cathodal F3 stimulation with respect to anodal F3/cathodal F4 and sham stimulations. In both males and females, instead, anodal F4/cathodal F3 stimulation negatively affected the tendency to adopt another’s point of view when the onlooker observed the actor reaching for the target object in the visual perspective taking task.

Previous neuromodulation studies suggested a preeminent role of the left DLPFC in processing facial expressions, although with some mixed results. In tasks requiring conscious or unconscious processing of emotional faces, Van Honk et al. [46] reported that low frequency, inhibitory rTMS over the right DLPFC increased selective attention to fearful faces, but only when facial expressions were consciously processed, likely due to enhanced activity of the contralateral, left prefrontal cortex. In a tDCS study, Vanderhasselt et al. [11] found that anodal stimulation over the left DLPFC enhanced performance for positive but not for negative faces in a task in which participants had to identify the word corresponding to or opposite to an emotional facial expression. In the same vein, Nitsche et al. [41] used bi-hemispheric tDCS and compared anodal, cathodal and sham stimulation over the left DLPFC during explicit identification of emotional faces. Results showed that identification of emotional faces was improved by anodal left tDCS stimulation; this improvement was greater for positive emotional faces. However, it is worth underlining here that Nitsche et al. [41] also found faster RTs under cathodal left tDCS with respect to sham stimulation; according to the authors the accompanying anodal right prefrontal tDCS might have contributed to this finding.

Before interpreting our data in light of the postulated role of lateral prefrontal cortex in emotional face processing, it is important to underline that bilateral tDCS over the DLPFC can also modulate response of the occipito-temporal cortex to recognition of non-emotional faces. In particular, Lafontaine et al. [42] showed that right anodal/left cathodal stimulation of DLPFC yielded faster recognition than the right cathodal/left anodal and sham conditions in a facial identity recognition task, consistent with a right hemisphere dominance for face processing [47]. These observations raise the possibility that our results showing a positive effect of anodal right/cathodal left stimulation on emotional expression recognition might be ascribed to a general facilitation for face processing. However, this post-hoc explanation would imply a generalized improvement of emotional expression recognition, independently from their valence, whereas it could hardly account for the specific effect of tDCS on the ability of male participants to recognize fearful expressions. Our results showing a selective effect of anodal right/cathodal left stimulation on fear recognition might be compatible with the valence theory of emotional processing [17], but we did not find that the reverse electrode arrangement (anodal left/cathodal right) influenced recognition of positive facial expressions, a pattern that would have strongly supported the model. Moreover, the valence theory does not make any prediction on gender related differences.

Gender related differential responses to tDCS-related have been reported in a recent study investigating the effects of tDCS applied over the superior temporal cortex in males and females during processing of facial expressions [32]. Results showed that females were significantly more accurate than males, independently from the stimulation condition, and that tDCS led to opposite effects on processing sad and happy faces in males and females. In our study females were significantly more accurate than males in recognizing all emotional faces, consistent with previous evidence [48–49], but only in males anodal right/cathodal left stimulation enhanced processing of threatening faces. Recently, Weisenbach et al. [28] observed that in recognizing fearful expression males and females activate similar regions, including frontal, parietal and temporal areas, but with different lateralization. Indeed, females generally demonstrated left-sided activation in frontal regions and right-sided activation in temporal regions, whereas males showed bilateral activations of frontal regions, but no temporal activation [28]. We might suggest that the prevalent involvement of frontal areas while processing threatening faces in males contributed to explain the effect of stimulation over DLPFC in males observed in the present study.

A relevant point of the present study is the different pattern of results elicited by stimulation over DLPFC in affective and cognitive tasks. Interestingly, we found that anodal right/cathodal left stimulation enhanced processing of fearful faces but interfered with performance on the cognitive task by reducing the onlooker’s tendency to adopt another individual’s point of view. Recently, Ramsey et al. [18] demonstrated that DLPFC respond during perspective judgments from both one’s own or another’s point of view, consistent with previous studies showing that the right lateral prefrontal cortex is involved in selecting the appropriate perspective and in inhibiting the irrelevant one [23]. However, other fMRI evidence revealed stronger activation of lateral prefrontal cortex for other- compared with self-perspective judgments [20], thus implying that prefrontal regions could differently respond to self and others’ point of view. In the present visual perspective experiment, participants were explicitly required to judge the visual scene from their own (self) or the actor’s (other) point of view. We found that, in the other-perspective, anodal right/cathodal left stimulation interfered with task performance, inhibiting the “altercentric tendency” and favoring one’s own viewpoint when participants observed the actor grasping the target object. Thus, we might suggest that such “enhanced egocentrism” can be related to increased inhibitory control of the dorsolateral prefrontal cortex over domain-specific neural systems involved in mental simulation of others’ actions, such as the mirror neuron system [50]. It has been suggested that sharing action representation via simulation may be a first step for more advanced mind-reading abilities [5], whereas higher level mentalizing implies further computations based on the ability to distinguish self from others’ perspective [51]. In this sense, mentalizing is closely related to cognitive control of the shared action simulation system; thus, mirroring of actions arises first, but subsequently a cognitive control mechanism has to come into play [52]. One possible speculative interpretation would imply that the “enhanced egocentrism” we found here might be related to inhibition of shared action representations induced by stimulation over the right DLPFC. Moreover, an alternative interpretation should be also taken into account. Indeed, considering the large size of the electrodes, and the proximity of stimulated DLPFC to a crucial node the action-observation system, i.e. the inferior frontal gyrus [50], one could conjecture that our electrode configuration exerted a direct inhibitory effect over the action simulation system via the cathodal stimulation in the left hemisphere, thus interfering with simulation of the actor’s action and reducing the tendency to adopt his viewpoint. Although disentangling between the two alternative interpretations needs a direct testing, we could maintain that inhibition of the action simulation system seems to prevent participants to adopt others’ perspective.

In conclusion, in the present study we found that anodal right/cathodal left stimulation over DLPFC can speed-up males’ responses to threatening faces whereas it interferes with the ability to adopt another’s viewpoint in both males and females. Although the small sample size might represent a limitation of the study, our sample was comparable to that used in previous studies investigating gender-related differences in response to tDCS [32,53]. Therefore, we believe that our results allow us to infer that stimulating activity of cognitive control areas can lead to different effects on social cognitive skills depending on the affective vs. cognitive nature of the task, and on the gender-related differences in neural organization of emotion processing. These findings fit neuropsychological and neurofunctional evidence showing that these two components can dissociate from each other [24–26], but our pattern of results is opposite to that previously reported in the TMS study by Kalbe et al. [26]. Future studies should clarify mechanisms sub serving the dynamic interplay between affective and cognitive mentalizing. In such studies it will be also important to understand whether and how individual differences in empathy or emotion recognition (beyond those related to gender) can influence neural and behavioral correlates of social cognition, and whether brain stimulation protocols can interact with behavioral training in shaping social interactions (see [54] for behavioral data on the effect of expertise on emotion recognition).

## Supporting Information

S1 File Dataset (in. xls format) containing all raw data for the Visual Perspective Taking task (VPT), and for the Recognition of Emotional Facial Expressions task (Emotions), in four separate worksheets. The file also includes a fifth worksheet containing all legends to raw data worksheets.
(ZIP)
